# Protocol for the process evaluation for a cluster randomised controlled trial evaluating primary school-based screening and intervention delivery for childhood anxiety problems

**DOI:** 10.1136/bmjopen-2023-082691

**Published:** 2025-02-20

**Authors:** Victoria Williamson, Michael Larkin, Tessa Reardon, Paul Stallard, Susan H Spence, Ian Macdonald, Obioha C Ukoumunne, Tamsin Ford, Mara Violato, Falko F Sniehotta, Jason Stainer, Alastair Gray, Paul Brown, Michelle Sancho, Fran Morgan, Bec Jasper, Lucy Taylor, Cathy Creswell

**Affiliations:** 1 Department of Experimental Psychology, University of Oxford, Oxford, UK; 2 Department of Psychiatry, University of Oxford, Oxford, UK; 3 Institute of Psychiatry, Psychology and Neuroscience, King’s College London, London, UK; 4 Institute for Health and Neurodevelopment, Aston University, Birmingham, UK; 5 University of Bath, Claverton Down, Bath, UK; 6 Australian Institute of Suicide Research and Prevention and School of Applied Psychology, Griffith University, Brisbane, Queensland, Australia; 7 Charlie Waller Trust, Newbury, UK; 8 NIHR ARC South West Peninsula, University of Exeter, Exeter, UK; 9 Department of Psychiatry, University of Cambridge, Cambridge, UK; 10 Health Economics Research Centre, Nuffield Department of Population Health, University of Oxford, Oxford, UK; 11 Division of Public Health, Social and Preventive Medicine, Centre for Preventive Medicine and Digital Health (CPD), Medical Faculty Mannheim, Heidelberg University, Heidelberg, Germany; 12 Stanley Primary School, Teddington, UK; 13 Bransgore C Of E Primary School, Christchurch, UK; 14 West Berkshire Council, Council Offices, Newbury, UK; 15 Square Peg (Team Square Peg CIC), Leamington Spa, UK; 16 PACT Parents and Carers Together CIC, Suffolk, UK

**Keywords:** Anxiety disorders, Child & adolescent psychiatry, QUALITATIVE RESEARCH

## Abstract

**Abstract:**

**Introduction:**

Anxiety problems are prevalent in childhood and, without intervention, can persist into adulthood. Effective evidence-based interventions for childhood anxiety disorders exist, specifically cognitive–behavioural therapy (CBT) in a range of formats. However, only a small proportion of children successfully access and receive treatment. Conducting mental health screening in schools and integrating evidence-based interventions for childhood anxiety problems may be an effective way to ensure support reaches children in need. The Identifying Child Anxiety Through Schools—Identification to Intervention (iCATS i2i) trial involves screening for childhood anxiety problems and offering a brief online parent-led CBT intervention. This paper presents the protocol for the process evaluation of the iCATS i2i trial, which aims to examine the implementation and acceptability of the study procedures, the mechanisms of change and whether any external factors had an impact on procedure engagement or delivery.

**Methods and analysis:**

This process evaluation will use both quantitative and qualitative methods to evaluate the implementation and acceptability of and barriers/facilitators to engagement and delivery of the iCATS screening/intervention procedures. Quantitative data sources will include opt-out and completion rates of baseline measures and usage analytics extracted from the online intervention platform. Qualitative interviews will be conducted with children, parents, school staff, iCATS i2i clinicians and researchers delivering study procedures. The Medical Research Council framework for process evaluations will guide study design and analysis.

**Ethics and dissemination:**

This study has received ethical approval from the University of Oxford Research Ethics Committee (R66068_RE003). Findings from the study will be disseminated via peer-reviewed publications in academic journals, conferences, digital and social media platforms and stakeholder meetings.

**Trial registration:**

ISRCTN76119074.

**STRENGTHS AND LIMITATIONS OF THIS STUDY**

- A strength of this study is the examination of acceptability and barriers/facilitators of Identifying Child Anxiety Through Schools—Identification to Intervention (iCATS i2i) via mixed method data collection from children, parents, school staff, iCATS i2i researchers and clinicians.
- A potential limitation is the majority of participants who opt-out or later dropout of iCATS i2i procedures may not participate in interviews, which could lead to a more positive overall evaluation of study procedures.
- The intervention will be delivered by English-speaking practitioners, which may unduly exclude participants who are not English speaking.

## Introduction

 Anxiety problems are among the most prevalent mental health problems in childhood and, without intervention, can often persist into adulthood.^1^ Cognitive–behavioural therapy (CBT) is an effective evidence-based intervention for childhood anxiety disorders^2^; however, only a very small proportion of children successfully access and receive treatment. For example, a recent study found that less than 3% of UK children with diagnoseable anxiety problems were able to access evidence-based treatments.^3^ Effective and efficient treatments for child anxiety problems now exist, such as parent-led CBT, that can facilitate early access to support.^4^ However, barriers to care are numerous,^5^ including a lack of help-seeking knowledge, stigma-related concerns^3 5^ and pressures on Child and Adolescent Mental Health Services (CAMHS), which means that they are often unable to meet the demand for non-urgent care.^6^

One promising way to address these barriers is to deliver interventions directly to parents through their children’s schools.^7^ While some universal schools-based interventions in schools show promise for some child outcomes,^8^ there are indicators that when those interventions are intended to improve mental health specifically^9^—rather than to improve indirect factors, such as health literacy,^10^ help-seeking^11^ or resilience^12^—a more targeted approach is likely to be required. One way to identify who interventions should target is through universal school-screening. This involves the administration of validated questionnaires to a year group (or entire school) to identify likely mental health problems.^13^ The implementation and uptake of school screening programmes are often low.^13 14^ Research has found that parents may be reluctant to engage with school-based mental health screening/intervention initiatives if they have previously felt blamed by them for their child’s difficulties or if they felt their child’s school had been unsupportive of their child’s mental health in the past.^15^ As such, prior to implementing a screening and intervention programme in schools, it is critical to establish whether the programme is acceptable; what barriers and facilitators to participation exist, whether any external factors impact delivery or engagement and which adaptations are needed to ensure the programme results in effective delivery and engagement.^16^

### The Identifying Child Anxiety Through Schools—Identification to Intervention (iCATS i2i) trial

Our proposed process evaluation is embedded within the iCATS i2i trial. This trial has involved the development of a brief screening tool for child anxiety problems, a codesign phase of work to develop procedures for delivering universal screening and targeted intervention,^17^ a feasibility study^7^ and a cluster randomised controlled trial (RCT).^18^ We include a brief summary of the cluster RCT here to provide context to the process evaluation design.

**Figure 1:**
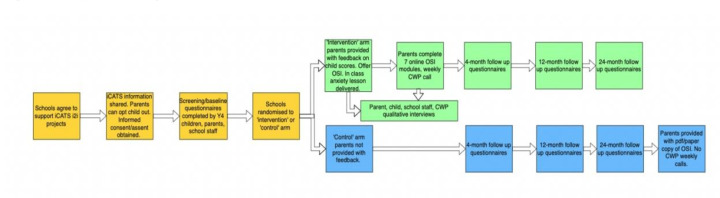
iCATS cluster randomised controlled trial procedures. Qualitative interviews (conducted after baseline and before 1-year follow-up) explore experiences of participation in the pathway, including screening and intervention. CWP, children’s Well-being practitioner; iCATS i2i, Identifying Child Anxiety Through Schools—Identification to Intervention; OSI, Online Support and Intervention for Child Anxiety; Y4, year 4.

In the main trial, participating schools (target 80 schools) across England have been randomised in a 1:1 ratio into one of two arms: the iCATS-i2i (intervention) arm and the usual school practice (control) arm. Full details on the trial procedures, including school randomisation process, can be found in Reardon *et al* and Ball *et al*’s study.^18 19^ The screening/intervention procedures in the iCATS i2i intervention arm consist of four key stages (see figure 1): (1) parent-report screening questionnaires for child anxiety problems are administered for all year 4 (Y4; aged 8–9 years) children; (2) screening questionnaires are scored by the research team to determine whether a child is likely to have anxiety problems; (3) feedback on questionnaire scores and likelihood of anxiety problems is provided to parents; (4) parents of children who screen ‘positive’ for likely anxiety problems are offered an online parent-led CBT intervention for child anxiety problems with telephone therapist support (Online Support and Intervention for Child Anxiety (OSI)); all parents (regardless of screening outcome) are given the opportunity to request OSI. OSI consists of seven online modules for parents who are supported by a weekly telephone call with a children’s well-being practitioner (CWP, National Health Service Band 5), with a follow-up telephone call 4 weeks later.^20^ OSI is only made available during the iCATS i2i trial to families in the intervention arm. Families in the treatment arm of the iCATS i2i trial who screen positive are actively offered treatment and those who screen negative can request the OSI treatment. Families in the usual school practice (control) arm do not receive feedback on questionnaire responses and are not offered OSI treatment—instead they can access whatever support is available as part of their ‘usual school practice’, as required. Usual school practice support for childhood anxiety varies somewhat across schools in the UK.^3 5 21^ We will systematically collect data on what usual school practice entails for all participating schools.

Children in the intervention arm schools are also provided with a whole class interactive lesson, which provides psychoeducation and information about coping strategies (problem-solving and help-seeking), and school staff are provided with information and resources about the OSI intervention.

For the purposes of the trial outcomes, participants are followed up at 4, 12 and 24 months post randomisation, using standardised questionnaire measures for quantitative evaluation (see Reardon *et al*,^18^ for details).

For the purposes of the process evaluation, qualitative interviews are also conducted with children, parents, school staff, iCATS researchers and CWPs/supervisors (target 55 interviews in total) to explore their experiences of the screening process and intervention procedures.

### Medical Research Council (MRC) guidelines

This process evaluation has been informed by the MRC advice on the process evaluation of complex interventions.^22^ The MRC guidance highlights three evaluation components—implementation, mechanisms of impact and context.

1. *Implementation*: an exploration of whether the intervention was delivered as intended (fidelity), the quantity of what was implemented (dose) and the ‘reach’ of the intervention’, as well as identifying any adaptations made.
2. *Mechanisms of impact*: an examination of the mechanisms through which an intervention brings about change by understanding how participants interact with the procedures.
3. *Context*: an exploration of factors external to the intervention, which may have affected the intervention’s acceptability, engagement or delivery (eg, home life for the family, school life for the child, comorbidities, COVID-19 social restrictions). MRC guidance suggests that researchers should relate contextual variations to a priori hypothesised causal mechanisms, or those arising from qualitative data analysis, to gain insights into context–mechanism–outcome patterns. In particular, this is likely to involve exploring differences between schools.

### The iCATS i2i process evaluations aims and objectives

Best practice in carrying out process evaluations is to outline the process evaluation methodology a priori.^23^ Using MRC guidelines and previous protocols of process evaluations as a guide,^24 25^ we outline our methodological approach and detail the planned process evaluation for the iCATS i2i trial. We include key questions that we will explore in the process evaluation, which are organised under the headings Implementation and acceptability, Mechanisms and Context, to be broadly consistent with MRC guidelines.^16 22^ While the MRC guidance for examining implementation often focuses on whether the intervention was delivered as intended in terms of fidelity, dose and reach,^22 26^ we will also focus on the acceptability of the implemented procedures given concerns about potential acceptability challenges identified in our previous iCATS i2i codesign work.^17^ We intend that this process evaluation will contribute towards the development of a set of transferable principles regarding school-based screening and intervention for mental ill-health more broadly, which could be offered in schools in the future.

Specific questions that will be addressed by this process evaluation are:

#### Implementation and acceptability

Key questions: Were the iCATS i2i screening/intervention procedures implemented as intended or were adaptations needed? Do the screening/intervention procedures reach children with anxiety problems? Are the screening/intervention procedures acceptable to schools and families? What is the variation in implementation and acceptability between schools and does variation relate to features of schools?

#### Mechanisms

Key questions: How do the screening/intervention procedures produce change? What barriers/facilitators to engagement and delivery exist? How could these potentially be overcome?

#### Context

Key questions: What—if any—external factors have an impact on iCATS i2i screening/intervention procedure engagement or delivery? Does context explain differences in outcomes or experiences between schools?

## Method

###  Ethical approval and dissemination

The iCATS i2i RCT has received ethical approval from the University of Oxford CUREC (R66068_RE003). Participant information sheets are provided to all potentially eligible participants prior to participation. Parents are given the opportunity to opt their child out of the research. Prior to providing any data, written informed consent is obtained from parents, teachers and qualitative interview participants, while children provide assent. Further information about trial procedures is available in full in the trial protocol.^18^ We will disseminate the findings in a number of ways, including at national/international conferences, in academic publications and funder reports.

### Patient and public involvement (PPI)

As detailed in our previous publications, the iCATS i2i procedures were codesigned in collaboration with extensive input from PPI.^17 27^

### Logic model

The MRC guidance on the development and evaluation of complex interventions notes that a key part of a process evaluation is to outline the processes of the intervention procedures and the outcomes it aims to attain using a logic model. The simplified logic model for the iCATSi2i screening/intervention procedures is shown in figure 1. Data collection and sources, as well as how these will address our process evaluation aims, can be found in online supplemental table 1.

### Overall design

This process evaluation will use a mixed methods design with purposively sampled qualitative data, supplemented by quantitative data from the trial, to strengthen our insights via triangulation. Quantitative data will include opt-out rates, completion rates for screening/baseline measures, feedback and support calls, online modules and time associated with OSI delivery (eg, time spent on feedback/support calls, online modules). Responses to a bespoke parent-report acceptability questionnaire, and routine measures collected within OSI (Session Rating Scale and Module Feedback Questionnaire), will also be used to assess the acceptability of procedures.

Qualitative data will include semistructured interviews conducted with children, parents, school staff, CWPs and research team members. Our intention is to create a comprehensive picture of families and schools’ experiences of the screening/intervention pathway procedures.

### Data collection procedure

Online supplemental table 1 illustrates the mapping between data sources and the questions which our evaluation will address.

### Quantitative data collection

Quantitative data collection is detailed in full in the trial protocol.^18^ Parents will have an opportunity to opt their child out of the research. When this does not happen, then parents, children and teachers will complete baseline questionnaires. For parents, the baseline assessment includes the 2-item child anxiety screening measure (iCATS-2) used in the screening/intervention procedures. School-level demographic information will be collected from publicly available information, and family-level demographic information will be collected from school records and parents.

CWPs and supervisors will complete activity logs to record completion and duration of feedback and OSI support calls, as well as supervision activities. OSI usage data (online module completion, completion of optional interactive activities within modules, time spent on module pages, number of times module pages are viewed) are collected within the OSI platform. Parents who use OSI complete measures built into each online module (including the Session Rating Scale and Module Feedback Questionnaire), and parents who complete screening questionnaires will be asked to complete a bespoke 7-item acceptability questionnaire to assess parent views of the procedures 4 months after randomisation.

### Qualitative data collection

The qualitative design is framed as a multiple perspective study,^28^ with inter-related subsamples. Interviews will be conducted with subsamples of parents (target n=20), children (target n=20) and school staff (target n=5) in the intervention arm, and with the CWPs and clinical psychologists facilitating the delivery of feedback and intervention (target n=5) and members of the research team who facilitated screening and data collection activities and delivered the anxiety lessons in schools (target n=5). This is a large total sample size for a qualitative study (total expected n=55), but it is necessary given the evaluative focus, and the need for diversity in the larger subsamples (parents, children). Interviews will be conducted during and after the feedback and intervention delivery period and will be completed prior to the 12-month follow-up. All interviews will be carried out by telephone or online video calling (Microsoft Teams) and audio recorded.

Parent, child and school staff will be purposively sampled with the aim of collecting data from a diverse cohort to include varying views on the screening/intervention trial programme. This approach will include ensuring perspectives from a range of socioeconomic, geographical location, gender and ethnicity backgrounds, and levels of interaction with OSI are included. We aim to collect interview data from families of children who screened ‘positive,’ screened ‘negative’, families who declined OSI and families who dropped out of OSI. We also aim to speak to participants in schools with higher rates of eligibility for free school meals, pupils with English as an additional language and parents opting out of the research. We anticipate that this sampling strategy will result in sufficient diversity to provide examples of both relatively poor and relatively good engagement with the iCATS i2i screening/intervention trial and allow for the identification of barriers and facilitators to implementation. School staff and parents who are participating in the ICATS i2i trial and who provided consent to take part in study interviews will be sent information about the opportunity to participate in interviews. Parents will be sent information about the opportunity for their child to take part in an interview.

Interview schedules will be informed by the research aims and existing literature on school-based screening/interventions for anxiety^14 17^ (online supplementalmaterial 1). To answer our study aims, interview questions will focus on what features of the iCATS i2i screening/intervention procedures worked well; whether any adaptations to procedures were needed; whether taking part was considered to be beneficial (or not) and why; whether any barriers to engagement or delivery were experienced; and if any external factors affected engagement/delivery.

### Data analysis

#### Quantitative data analysis

To assess reach and acceptability of procedures, we will investigate participation rates in each element of the screening/intervention procedures. This will include examining the number and proportion of (1) parent opt-outs, (2) completed screening questionnaires (parent-report iCATS-2), (3) screen positives (child scores 3–6 on parent-report iCATS-2) among all eligible Y4 children. The number and proportion of completed (4) feedback calls with a CWP, (5) online modules and support calls (separately for each module) and (6) core intervention content (first five modules) will be examined for both screen positives and all eligible Y4 children. We will also examine the number and proportion of completed baseline measures for eligible Y4 children (coded as yes, no, partial) for each reporter (parent, child, teacher). To further assess engagement with and delivery of OSI, descriptive statistics will be used to summarise the following among parents who start OSI: completion of optional questions/activities within online modules, time (minutes) spent on online modules, number of times online module pages are viewed, time (minutes) spent on support calls, CWP/clinical psychologist time (minutes) spent on associated administrative and supervision activities. Responses to the parent-report acceptability questionnaire, the Session Rating Scale and Module Feedback Questionnaire, will also be summarised using descriptive statistics.

To explore factors that may influence engagement with the screening/intervention procedures, we will examine participation rates among schools with above/below average proportion of pupils eligible for free school meals and above/below average proportion of pupils with English as an additional language and examine school and family-level characteristics associated with completion of the OSI online modules, feedback and OSI support calls, core intervention content, time spent on online modules, number of times online module pages are viewed, time spent on support calls and associated administrative/supervision activities.

#### Qualitative data analysis

Qualitative interviews will be transcribed verbatim, with identifying personal information removed on transcription. Transcripts will be checked against audio recordings, and then audio recordings will be destroyed. Transcripts will be imported into Nvivo V.12 to facilitate data management. Reporting of qualitative findings will follow the Consolidated criteria for Reporting Qualitative research checklist.^29^

A subset of the transcripts will be analysed separately first to create a coding template which will cover how the screening/intervention procedures are experienced in the context of participant’s distinctive lives. This analysis will be used to develop a template framework. All transcripts will then be analysed against this framework using template analysis, with modifications to the template made after careful consideration of each transcript.^30^ We expect the developed template will include: what aspects of the screening/intervention procedures were acceptable; if any adaptations to the pathway procedures were needed; barriers or facilitators to engagement and delivery; and whether any external factors impacted the engagement or delivery of the procedures.

#### Integration of data analysis

The qualitative and quantitative data will be analysed separately and then mixed during analysis for triangulation to provide a more complete picture, as described below.^31^ The quantitative and qualitative strands will play an equally significant role in addressing the process evaluation research questions. A triangulation protocol will be followed^32^ involving:

1. Sorting findings from the qualitative and quantitative datasets into categories or ‘meta-themes’ that address the research questions to determine overlap and divergence.
2. Comparing findings from the data sources using a convergence coding scheme to determine the degree and type of convergence within category or theme areas. Researchers will consider if there is agreement, partial agreement, silence or dissonance between findings from different datasets. ‘Silence’ is where a finding that arises from one dataset is not found in another and can help with the interpretation of the results and lead to further investigations.^32^
3. Reviewing all meta-themes to assess the level of convergence and where/when researchers have different perspectives of the findings.
4. Multiple researchers (VW, TR, CC, ML) will examine the set of findings to clarify the interpretation and determine the level of agreement among researchers. Disagreements will be managed by re-examining the data as a group, with final decisions made by CC and ML.

The process evaluation data will be analysed independently of the main trial clinical and cost-effectiveness outcomes. The statisticians (OU, SB) and health economist (MV) conducting the main trial quantitative data analysis^18^ will be unaware of the findings from the process evaluation until the primary and secondary clinical and health economic outcomes have been analysed. The combined quantitative and qualitative data in the process evaluation is expected to help develop an in-depth understanding of the main trial outcomes.

### Rigour and reliability

This process evaluation will be conducted by a team of experienced researchers with considerable expertise of both mixed methods and undertaking large-scale intervention trials for childhood anxiety disorders. Several steps will be taken to ensure a rigorous approach to data collection and analysis: (1) cluster (school) and purposive sampling will be conducted for qualitative interviews to ensure a broad and diverse sample and, thus, fair conceptual transferability; (2) data collection and analysis will follow a systematic approach, including a range of both qualitative and quantitative data; (3) researchers will reflect on their role and input in data generation and analysis; (4) credibility checking will be conducted through reflective discussions with coauthors and a small expert reference group; (5) results will be triangulated across several sources of data; and (6) ‘sensitivity to context’ will be considered by incorporating relevant literature and theory as well as examining differing perspectives and the context in which data and results have been generated.^33^

## Discussion

This article outlines the rationale, design and methodological approach for the mixed methods process evaluation of the iCATSi2i screening and intervention procedures for children with anxiety problems. The process evaluation is designed to examine whether the screening/intervention procedures are implemented as intended or if adaptions are needed; if procedures are acceptable to schools and families; how the screening/intervention procedures produce change; whether barriers/facilitators to engagement and delivery exist; and whether any external factors impact procedure engagement or delivery. By detailing our process evaluation approach, as informed by the MRC guidelines,^22^ this article not only adds to the literature on process evaluation protocols with a mixed methods design but will also improve the integrity of our process evaluation and overall RCT quality.^23 34^

### Strengths and challenges

It is anticipated that actively combining both qualitative and quantitative data in the process evaluation will help us to better understand and interpret the overall iCATS i2i trial outcome data. For example, by comprehensively examining whether the iCATS i2i screening/intervention procedures were adhered to and acceptable and the contexts surrounding that, this process evaluation will help determine both potential positive and negative aspects of the iCATS i2i procedures. If some negative outcomes are found from using the iCATS i2i screening/intervention procedures, the process evaluation will be a beneficial resource to determine whether a failure of procedure implementation occurred and if this was due to, for example, factors associated with participants’ experiences or circumstances (eg, lack of motivation or resources, beliefs about online interventions, etc). This could potentially help with future implementations of iCATS i2i, if indicated, and also help inform the development and implementation of wider school-based screening and intervention programmes aimed at supporting children with mental health problems.

Collecting data from a range of participants (ie, children, parents, teachers, researchers and CWPs) using multiple methods will produce a nuanced understanding of the mechanisms contributing towards the experience of the iCATS i2i procedures. Including teachers, children and parent report measures may also provide data about the acceptability of carrying out such screening procedures, which would be beneficial beyond the iCATS i2i study and inform future screening/intervention trials. Moreover, the target sample size for qualitative interviews (n=55) and inclusive sampling approach (eg, conducting interviews with screen ‘positive’ as well as ‘negative’ families, families who dropout of OSI, etc) is expected to be adequate to capture a range of perspectives, providing rich detailed data.

One potential limitation that may arise is that the majority of participants who opt-out or later dropout of the iCATS i2i procedures are more likely to decline to complete interviews, which could lead to a more positive overall evaluation of the procedures. We will attempt to overcome this by making a concerted effort to recruit parents who dropout of or choose not to take up OSI or, if this is not possible, those who complete fewer OSI modules to interviews. Second, while we will be able to provide translated copies of the information sheets, OSI will be delivered by English-speaking CWPs for practical reasons and this may unduly exclude parents who are not English speaking. Third, it is possible wider trial research activities influence engagement with the screening/intervention procedures in ways that would not apply if the procedures were to be implemented in practice. For example, the screening questionnaire is a 2-item parent-report measure, but in the trial parents, children and teachers also each complete a number of measures to assess secondary trial outcomes. In addition, the team of researchers with responsibility for conducting this process evaluation will also be involved in carrying out the trial procedures and some will be involved in conducting the trial outcome analysis. This integration will help facilitate data sharing, but there is potential for bias in the interpretation of procedure functioning to arise. A reflective approach to data collection and analysis will be employed to improve the reliability and validity of the findings.

The iCATS i2i screening/intervention procedures are complex and involve a range of inter-related components and multiple stakeholders. There may be some differences in procedure implementation across schools and there is likely to be adaption to and learning from the procedures as delivery proceeds.^35 36^ Moreover, given that schools and families are each unique and complex ecosystems where a community of individuals interact and coexist, school and family contexts cannot be considered ‘static’. We will need to recognise that the iCATS i2i procedures are being delivered within shifting environments, and the rolling out of the iCATS i2i school screening/intervention procedures may also have some influence on the environment. There may be considerable challenges in monitoring and precisely assessing the various iCATS i2i procedure implementation processes, components and changing environments and how they relate to outcomes. It is hoped that by including ‘adaptations’ as a core aim in our process evaluation, that any necessary departures from study procedures are recognised and captured. Overall, this process evaluation is expected to further our understanding of the acceptability of screening/intervention procedures for child anxiety problems in a school context to inform future efforts to address child mental health problems.

### Trial status

Recruitment of participants is ongoing.

## supplementary material

10.1136/bmjopen-2023-082691 online supplemental file 1

10.1136/bmjopen-2023-082691 online supplemental file 2
