# Supplementary material for: Protocol for the process evaluation for a cluster randomised controlled trial evaluating primary school-based screening and intervention delivery for childhood anxiety problems
Source: BMJ Open. 2025 Feb 20;15(2):e082691. doi: 10.1136/bmjopen-2023-082691 (PMC11842992; doi:10.1136/bmjopen-2023-082691)
Supplement: online supplemental file 1 [file bmjopen-15-2-s001.pdf]

Supplementary Material 1, Table 1

		**Explanatory data**				**Key Questions**					
**Participants**	**Mode of data collection**	**Data outcomes**	**Timepoint**	**Number/ frequency**	**Trial arm**	**Were the screening/intervention procedures implemented as intended or were adaptations needed?**	**Do the screening/intervention procedures reach children with anxiety problems?**	**Are the screening/intervention procedures acceptable?**	**How do the screening/intervention procedures produce change?**	**What barriers/facilitators to engagement with and delivery of the screening/intervention procedures exist?**	**What - if any - external factors impact screening/intervention engagement or delivery?**
Y4 children	Questionnaires	Completion of baseline measures	Baseline	Baseline (all Y4)	Both		X	X			
	Interview	Experience of being involved in the screening/intervention pathway, including anxiety lesson	After baseline, before 1 year follow up.	20 interviews	Intervention	X	X	X	X	X	X
Y4 parents	Questionnaires	Opt-out rates, completion of screening and baseline measures, screen positive rates	Baseline	Baseline (all Y4)	Both		X	X		X	X
	Interview	Experience of being involved in the screening/intervention pathway	After baseline, before 1 year follow up	20 interviews	Intervention	X	X	X	X	X	X
	Questionnaires	Bespoke acceptability questionnaire	4 month follow-up	4 month-follow-up for all parents who complete screening questionnaires	Intervention arm			X		X	
	OSI usage	Completion of online modules and online module activities, time spent on each module and number of times	Throughout OSI delivery	Data collected for all parents who use OSI	Intervention	X	X	X			

		module pages are viewed									
	Questionnaire measures to guide future OSI developments	Session Rating Scale<br>Module Feedback Questionnaire	8 online modules (Module 0 to Follow-up)	Data collected for all parents who use OSI	Intervention arm			X		X	
Y4 teachers & school staff	Questionnaires	Completion of baseline measures	Baseline	Baseline (for all Y4)	Both			X			
	Interview	Experience of being involved in the screening/intervention pathway	After baseline, before 1 year follow up	5 interviews	Intervention	X	X	X	X	X	X
CWPs/supervisors	Interview	Experience of delivering feedback and OSI to families	Throughout feedback and intervention delivery	5 interviews	Intervention	X		X	X	X	X
	CWP-parent contact time and supervision time	Completion of feedback and support calls, time spent on calls and supervision activities	Throughout feedback and intervention delivery	Data collected for all parents who use OSI	Interview	X	X	X		X	X
iCATS research team	Interview	Experience of delivering screening/intervention activities	After baseline, before 1 year follow up.	5 interviews	Both	X	X	X	X	X	X

Table 1. Relationship between process evaluation questions, explanatory data, data sources and outcomes

Note: Qualitative interviews (N=55) will be conducted during and after the intervention delivery period and will be completed prior to the 12-month follow-up. CWP = children's wellbeing practitioner. OSI = online support and intervention. Y4 = year four.
