# Supplementary material for: Protocol for the process evaluation for a cluster randomised controlled trial evaluating primary school-based screening and intervention delivery for childhood anxiety problems
Source: BMJ Open. 2025 Feb 20;15(2):e082691. doi: 10.1136/bmjopen-2023-082691 (PMC11842992; doi:10.1136/bmjopen-2023-082691)
Supplement: online supplemental file 2 [file bmjopen-15-2-s002.pdf]

Supplementary Material 1

**Indicative guide for in-depth interviews with parents**

<u>Topics to explore in the interview</u>

- How have you found taking part in the iCATS study so far?
    - Were there any issues or concerns you were hoping iCATS would help with?
    - Did you have any concerns or worries that made you hesitant to get involved?
    - Was there anything you think could have been done to encourage you/others to get involved?
- How did you get on with the initial questionnaires and consent forms?
    - Did you fill these in or did your child's other parent? Why was this?
    - Was there anything that you found difficult in filling in the questionnaires?
    - Was there anything that could've been made easier for you here?
    - How did you find accessing these online (or by paper)?
    - How did you feel about how your data was being managed/stored? What was important for you here?
    - How did you feel about taking part being opt out?
- How did your child get on with these questionnaires?
    - Did they do the questionnaire at home with you or at school? What did you think about this approach?
    - What do you think about this study looking at anxiety in Y4 as an age group?
- Was there anything you feel you or your child gained or learnt from filling in the questionnaires?
- How did you feel about your child's teacher also filling in a questionnaire about your child?
- How did you find the feedback about your responses to the questions about your child's fears and worries?
    - Did you have any concerns at this stage?
    - Was there any more information you would have liked to have had?
- Did the feedback you received on the questionnaires affect how you felt about doing the OSI intervention?
- How did you find accessing OSI?
    - What did you think about everything being online/remote?
    - How do you think this would compare to a F2F course?
    - When do you find time to work through OSI?
    - How did you decide which parent would do OSI?
    - How did you find doing the activities with your child?
- What impact do you think the activities have had on their fears and worries?
    - How do you feel about managing your child's difficulties with fears and worries having done OSI?
    - Has your knowledge or confidence in supporting your child changed following OSI?
    - Has there been any changes in your family life since taking up OSI?
    - Has doing OSI had any impact on your own wellbeing?
    - Have you become aware of any new sources of support as a result of being part of OSI?
- What did you think of the weekly phone calls?
    - How do you feel about the number or length of sessions?
    - How have you found the 1 month break?
        - OR How do you feel about there being a 1 month break?
    - How do you feel about your child's 'discharge' letter?
        - Will you share this with your child's school? Why or why not?
    - In an ideal world, is there any other support or help you would've liked to receive?
    - Could anything have been made easier for you/others to keep engaging with OSI?

- Have you spoken to or interacted with your child’s school about iCATS?
    - What was this experience like?
    - Could anything have been improved here?
    - Do you think a parent-school conversation is needed? Or is this not necessary?
- Have you spoken with other people about iCATS?
    - Have you spoken to any parents who dropped out of or chose not to take part in iCATS? Do you know why they made this decision?
    - After finishing OSI do you think you will speak to other people about it?
- Is there anything we can do to make sure iCATS works well for other families in future?
- How would you describe your child’s school culture or attitude towards mental health or anxiety?
    - Do you think iCATS may have any broader effects on your child’s school or your community?
    - For those families who have a difficult relationship with their child’s school, what impact on the parent-school do you think running iCATS may have?

**Indicative guide for in-depth interviews with school staff**

Topics to explore in the interview

- How have you found being part of the iCATS study so far?
- What made you and your school want to get involved?
  - Were there any particular motivators for your school to want to join in?
  - Are there any factors that made you or your school hesitant to take part?
  - Was there anything we could have done differently to encourage your school (or other schools) to get involved?
- What did you hope you/your pupils/your school would get out of taking part in iCATS?
- How did you get on with the initial questionnaires and consent forms?
  - What did you think about the number/length of questionnaires?
  - How did you find accessing these online?
  - Did you have dedicated time to fill them in?
  - Is there is anything you feel you learned from filling in the questionnaires?
  - How did you feel about how your data was managed/kept secure? What was important for you here?
  - Did filling in the questionnaires have any impact on your knowledge or confidence in supporting children in your class?
  - Was there anything that could've been made easier for you here?
- How did your pupils get on with their questionnaires?
  - Did they do the questionnaire at home or at school? What did you think about this approach?
  - Did they need any help to fill them in?
  - Was there anything that could have been done differently here?
- Do you know how any of your class's parents got on with filling in their initial questionnaires?
  - Did any parents have any difficulties accessing or filling them in?
  - Why may some parents have a tough time filling in the questionnaires?
  - Was there anything we could do to support parents better during this process?
- Did you see the feedback about pupils' scores?
  - Did you see the list of the pupils who screened 'positive'?
    - If no, why was this? Would you have liked to see it?
  - Were the outcomes what you were expecting?
  - Initially we planned for the school iCATS lead to give this feedback, how do you feel about the feedback coming from the research team instead?
  - Could anything have been done differently here?
- What did you think about the Y4 anxiety lesson?
  - Was there anything that was difficult to understand?
- Did taking part in iCATS and/or any of the information we have shared make any differences to how you feel you manage anxiety or other problems within the classroom? If so, in what way?
- Have you spoken to or interacted with your pupils or parents about their experience of iCATS?
  - What was this experience like?
  - Did you get asked any questions by pupils/parents? How did this go?
  - Did you speak with any parents/pupils who didn't want to take part or dropped out? What seemed to contribute towards this?
  - Did you speak to any parents that received the online intervention? How did they get on?
  - Did you speak to any parents that were offered OSI who didn't take it up? What have their experiences been?

- Have you spoken with other people about iCATS? (e.g. colleagues, your own friends/family). What have their reactions been?
- Have you had any situations or instances that stand out to you about how people have understood what we're doing with iCATS?
- What do you think about iCATS being for Y4 children? How does this fit with existing school procedures (e.g. exams in Y5)?
- How would you describe your school's culture or attitude towards child mental health or anxiety?
    - Do you think iCATS has had or may have any broader effects on your school or your community?
    - For those families who have a more difficult/strained relationship with their child's school, what impact do you think iCATS could have on that parent-school relationship?
- Is there anything we can do to make sure iCATS works well for other schools or families in future?

**Indicative guide in-depth interviews with Y4 children**

Topics to explore in the interview

- What did you think about the iCATS when iCATS was first talked about at your school?
- Was there any more information you would have liked to know about iCATS before joining in?
    - What did you think about filling in the questionnaire about your fears and worries?
    - Did you do the questionnaire at home or at school?
    - Did you do the questionnaire in big groups or small groups?
    - Did you learn anything from filling in the questionnaire?
    - Could anything have been done differently to make filling in the questionnaire easier for you?
- What did you think about the lesson on fears and worries?
    - What bits did you like about the lesson?
    - What bits did you not like?
    - What did you think about the strategies it explained for what to do when you are worried?
    - Have you used any of the strategies?
- What did you think about your parent(s) doing the course to help you with your fears and worries?
    - How did doing the iCATS activities with your parent(s) make you feel?
    - Were there any activities you found really fun?
    - Were any activities hard? Why do you think that was?
- How did you find using the Monster's Journey game?
- Did you speak to anyone (e.g. friends, family, teachers) about your parents doing the course to help you with your fears and worries?
    - What did you say? How did they respond?
    - If you didn't speak to anyone, why was this?
- Do you think your parents doing the lessons about your fears and worries had any impact on other members of your family or how your family gets along?
    - Why or why not?
- Is there any extra help for your fears or worries that you would have liked to have?
- Are there any other thoughts you have about the iCATS project that we should know?

**Indicative guide for in-depth interviews with iCATS researchers**

<u>Topics to explore in the interview</u>

- Have you been involved in any iCATS i2i school recruitment?
  - What has encouraged schools to get involved?
  - Are there any barriers to school's getting involved?
  - What sorts of questions or concerns do school's have before signing up?
- How have you found interacting with school staff?
  - What factors would you say make for an 'engaged' school?
  - What does an 'engaged' school look like?
  - What factors would you say make a school more difficult to interact or engage with?
  - What does a 'not engaged' school look like?
  - Can anything be done to improve school engagement? How?
- How have you found working with iCATS school leads?
  - How have you found working with school staff?
  - What questions/concerns do school staff typically have?
- How have you found interacting with iCATS parents?
  - What sorts of questions/concerns do parents usually have?
  - Have you spoken to any parents who dropped out of the study? What were their reasons?
- How have you found doing the questionnaire administration and data collection?
  - Did you send out and collect parent questionnaires?
    - Have you had to support any parents in filling these in?
    - What sort of support did parents need?
  - Did you go to schools and help administer child questionnaires?
    - What was this like?
    - What things are needed for this to go well?
  - Did you help any teachers to do their questionnaires?
    - What sort of support did they need?
- How did you find being part of the Y4 child anxiety lesson?
  - Did you help to deliver this in a school?
  - What factors are important in making the lesson go well?
  - What things can mean the lesson doesn't get delivered well?
  - Do you think running the anxiety lesson has any broader impacts in schools?
- Have you had any situations or instances that stand out to you about how people have understood what we're doing with iCATS?
- How would you describe the general climate/culture with regard to mental health in the schools that you visited?
- What do you think cuts through to schools most clearly, in terms of the appeal/advantages of iCATs?
  - What about to families?

**Indicative guide for in-depth interviews with iCATS CWPs and clinical psychologists.**

Topics to explore in the interview

- How have you found the feedback calls with parents/carers in in the iCATS-i2i trial?
    - How did the feedback we provided families on the questionnaire responses seem to affect how parents/carers felt about doing OSI?
    - Have parents/carers raised any concerns related to the feedback they received?
    - What are your thoughts on the feedback coming from the research team, rather than the school?
    - Have you had any calls with parents/carers who did not complete the initial questionnaires?
    - Could anything have been done differently in how we provide feedback to families and offer OSI?
    - Was there any more information parents/carers would have liked to have had?
    - Are there any changes you think could be made to how we share feedback with families/how we offer OSI?
- How have you found delivering [and/or supervising the delivery of] OSI?
- What did you think about everything being online/remotely?
    - Did the online/remote delivery present challenges for you? And for parents? How did you try to manage these challenges and what worked/worked less well and why?
    - Did the online/remote delivery bring any benefits for you? And for parents?
- What impact do you think OSI has had on children's fears and worries?
- What impact do you think OSI has had on other aspects of family life?
- From your experience of working with parents, do you think OSI and iCATS more generally has had any impacts on the environment within participating schools or classes?
- How do you feel about the structure of the OSI programme? E.g. the number and length of online modules, the number and length of support calls, the 1 month follow-up
- How do you find keeping to the OSI guidance when supporting families?
    - Are all calls with parents generally the same or do some differ?
    - How do you manage this?
- Have you had contact with school staff or other professionals about families who received OSI? How have you found that?
- Are there any changes you think that we need to make to OSI for future delivery through primary schools?
