# Supplementary material for: Protocol for the process evaluation for a cluster randomised controlled trial evaluating primary school-based screening and intervention delivery for childhood anxiety problems
Source: BMJ Open. 2025 Feb 20;15(2):e082691. doi: 10.1136/bmjopen-2023-082691 (PMC11842992; doi:10.1136/bmjopen-2023-082691)
Supplement: Supplementary file 3 [file bmjopen-2023-082691.reviewer_comments.pdf]

PEER REVIEW HISTORY

BMJ Open publishes all reviews undertaken for accepted manuscripts. Reviewers are asked to complete a checklist review form and are provided with free text boxes to elaborate on their assessment. These free text comments are reproduced below.

ARTICLE DETAILS

**TITLE (PROVISIONAL)**	Protocol for the process evaluation for a cluster randomised controlled trial evaluating primary school-based screening and intervention delivery for childhood anxiety problems
**AUTHORS**	Williamson, Victoria; Larkin, Michael; Reardon, Tessa; Stallard, Paul; Spence, Susan; Macdonald, Ian; Ukoumunne, Obioha; Ford, Tamsin; Violato, Mara; Sniehotta, Falko F.; Stainer, Jason; Gray, Alastair; Brown, Paul; Sancho, Michelle; Jasper, Bec; Taylor, Lucy; Creswell, Cathy; Morgan, Fran

VERSION 1 - REVIEW

**REVIEWER NAME**	Kaar, Jill
**REVIEWER AFFILIATION**	University of Colorado - Anschutz Medical Campus
**REVIEWER CONFLICT OF INTEREST**	None
**DATE REVIEW RETURNED**	16-Jan-2024

**GENERAL COMMENTS**	This manuscript documents a protocol for the school-based anxiety intervention referred to as Identifying Child Anxiety Through Schools - Identification to Intervention (iCATS i2i).<br><br>Thus far iCATS i2i has included:<br>1) Development of brief screening tool for children anxiety problems<br>2) Co-design process to develop procedure to universal screening<br>3) Feasibility study<br>4) Cluster RCT<br><br>The iCATS i2i program is an online parent-led CBT intervention with telephone therapist support for children with anxiety problems. The program consists of 7 online modules for parents and include weekly telephone calls with a children's wellbeing practitioner.<br><br>The RCT takes place in 80 schools across England and have been randomized to 1) iCATS i2i or 2) Usual practice.<br>Treatment includes: iCATS i2i online program for parents; whole class interactive lessons on psycho-education and information about coping strategies and school staff are provided with info on OSI intervention.<br>Usual care includes: xxx<br><br>iCATS i2i is delivered in four stages<br>1) Parent-report screening for students aged 8-9 years<br>2) Screening responses scored and research team categorizes students by anxiety problems (yes/no)<br>3) Parents receive scores<br>4) Parents of students who screen positive are offered program<br><br>Timing: baseline, 4, 12, 24 months post.

	Questions:<br>1) Is OSI and iCATS i2i the same?<br>2) Page 10/46 lines 47-51 states: parents of students with negative screen are given "opportunity to request" program; and on page 11/46 lines 1-10 states "not offered" program. Please clarify.<br>3) What is schools usual care for kids with anxiety?<br>4) How are schools randomized?<br>5) What is consent process?<br>6) How is the assessment completed?<br>What are the assessments? Where is the info on the developed anxiety scale?

**REVIEWER NAME**	Shinde, Sachin
**REVIEWER AFFILIATION**	Harvard University
**REVIEWER CONFLICT OF INTEREST**	None
**DATE REVIEW RETURNED**	01-Feb-2024

**GENERAL COMMENTS**	Thank you for this opportunity to review this well-written manuscript outlining the process evaluation protocol for assessing the implementation, acceptability, and factors influencing engagement and delivery of iCATS screening/intervention procedures. I have a few minor suggestions for your consideration:<br><br>Abstract: Including the objectives of the process evaluation in the abstract could enhance its readability.<br><br>Introduction: It could be beneficial to mention specific barriers to care.<br><br>Methods: Providing additional information on how participants for qualitative interviews will be recruited would be helpful.

VERSION 1 – AUTHOR RESPONSE

Reviewer 1

1) Is OSI and iCATS i2i the same?

No, OSI is the treatment offered to parents in the intervention arm as part of the iCATS i2i programme.

2) Page 10/46 lines 47-51 states: parents of students with negative screen are given "opportunity to request" program; and on page 11/46 lines 1-10 states "not offered" program. Please clarify.

As stated on page 11, families in the treatment as usual control arm of the trial are not offered the treatment. Families in the treatment arm of the trial who screen positive are actively offered treatment and those that screen negative can request treatment. We have added a statement on page 8 to make this distinction clearer.

OSI is only made available during the trial to families in the intervention arm. Families in the treatment arm of the trial who screen positive are actively offered treatment and those that screen negative can request treatment. Families in the usual school practice (control) arm do not receive feedback on questionnaire responses and are not offered OSI – instead they can access whatever support is available as part of their ‘usual school practice,’ as required.

3) What is schools usual care for kids with anxiety?

Usual care for anxiety difficulties can differ between schools across the UK. We have added information about the available support options and references for interested readers on page 8:

Families in the usual school practice (control) arm do not receive feedback on questionnaire responses and are not offered OSI – instead they can access whatever support is available as part of their ‘usual school practice,’ as required. Usual school practice support for childhood anxiety varies somewhat across schools in the UK[3,5,20]. We will systematically collect data on what usual school practice entails for all participating schools.

4) How are schools randomized?

On page 7 we state that ‘participating schools…are randomised in a 1:1 ratio into one of two arms’. The protocol for the clinical and economic aspects of the trial has been published here and contains information on the randomisation methods: https://link.springer.com/article/10.1186/s13063-022-06773-0 . We have cited this trial protocol for interested readers. As such we have not added any further detail in the present paper as this particular protocol focusses on the process evaluation.

5) What is consent process?

We detail the consent process on page 10 and we have added a statement for interested readers that further information about the conduct of the trial – including the consent process – is available in the full trial protocol.

Ethical approval and dissemination
The iCATS i2i RCT has received ethical approval from the University of Oxford CUREC (R66068_RE003). Participant information sheets are provided to all potentially eligible participants prior to participation. Parents are given the opportunity to opt their child out of the research. Prior to providing any data, written informed consent is obtained from parents, teachers, and qualitative interview participants, and children provide assent. Further information about trial procedures is available in full in the trial protocol [18]. We will disseminate the findings in a number of ways, including at national/international conferences, in academic publications and funder reports.

6) How is the assessment completed? What are the assessments? Where is the info on the developed anxiety scale?

Due to journal word limit restrictions, we are not able to provide a full detailed list of all psychometric questionnaires used in the present study protocol and we should also stress that the aim of this article is to describe the process evaluation procedures rather than the trial itself. Full details of the wider trial procedures are provided in the trial protocol as noted above. However, we provide complete details about where this information can be accessed by interested readers in this process evaluation

protocol on page 12:

Quantitative data collection is detailed in full in the trial protocol [18]. Parents will have an opportunity to opt their child out of the research. When this does not happen, then parents, children and teachers will complete baseline questionnaires. For parents, the baseline assessment includes the 2-item child anxiety screening measure (iCATS-2) used in the screening/intervention procedures. School-level demographic information will be collected from publicly available information, and family-level demographic information will be collected from school records and parents.

Reviewer 2

1. Abstract: Including the objectives of the process evaluation in the abstract could enhance its readability.

We thank the reviewer for this suggestion and have added a statement in our abstract outlining our objectives.

Introduction: Anxiety problems are prevalent in childhood and, without intervention, can persist into adulthood. Effective evidence-based interventions for childhood anxiety disorders exist, specifically cognitive behavioural therapy (CBT) in a range of formats. However, only a small proportion of children successfully access and receive treatment. Conducting mental health screening in schools and integrating evidence-based interventions for childhood anxiety problems may be an effective way to ensure support reaches children in need. The Identifying Child Anxiety Through Schools – Identification to Intervention (iCATS i2i) programme involves screening for childhood anxiety problems and offering a brief online parent-led CBT intervention. This paper presents the protocol for the process evaluation of the iCATS i2i programme which aims to examine the implementation and acceptability of the study procedures, the mechanisms of change and whether any external factors had an impact on procedure engagement or delivery.

2. Introduction: It could be beneficial to mention specific barriers to care.

We have added information about barriers to care in our introduction on page 6, with references cited for interested readers:

Effective and efficient treatments for child anxiety problems now exist, such as parent-led CBT, that can facilitate early access to support [4]. However, barriers to care are numerous [5], including a lack of help-seeking knowledge and stigma-related concerns [3,5], and pressures on Child and Adolescent Mental Health Services (CAMHS) which means that they are often unable to meet the demand for non-urgent care [6].

3. Methods: Providing additional information on how participants for qualitative interviews will be recruited would be helpful.

We have provided more information about the recruitment of participants to qualitative interviews on pages 13 and 14:

Parent, child and school staff will be purposively sampled with the aim of collecting data from a diverse cohort to include varying views on the screening/intervention programme. This approach will

include ensuring perspectives from a range of socio-economic, geographical location, gender and ethnicity backgrounds, and levels of interaction with OSI are included. We aim to collect interview data from families of children who screened ‘positive,’ screened ‘negative’, families who declined OSI, and families who dropped out of OSI. We also aim to speak to participants in schools with higher rates of eligibility for free school meals, pupils with English as an additional language, and parents opting out of the research. We anticipate that this sampling strategy will result in sufficient diversity to provide examples of both relatively poor and relatively good engagement with the iCATS i2i screening/intervention programme and allow for the identification of barriers and facilitators to programme implementation. School staff and parents who are participating in the ICATS i2i trial and who provided consent to take part in study interviews will be sent information about the opportunity to participate in interviews. Parents will be sent information about the opportunity for their child to take part in an interview.
